# Histopathological Changes of the Thyroid and Parathyroid Glands in HIV-Infected Patients

**DOI:** 10.1155/2014/364146

**Published:** 2014-01-22

**Authors:** Rabia Cherqaoui, K. M. Mohamed Shakir, Babak Shokrani, Sujay Madduri, Faria Farhat, Vinod Mody

**Affiliations:** ^1^Howard University Hospital, 2041 Georgia Avenue NW, Washington, DC 20060, USA; ^2^Department of Endocrinology, National Naval Medical Center, 8901 Wisconsin Avenue, Bethesda, MD 20889, USA; ^3^Department of Pathology, 2041 Georgia Avenue NW, Washington, DC 20060, USA; ^4^Division of Endocrinology and Metabolism, 2041 Georgia Avenue NW, Washington, DC 20060, USA; ^5^Division of Infectious Disease, Department of Internal Medicine, Howard University Hospital, 2041 Georgia Avenue NW, Washington, DC 20060, USA

## Abstract

*Objective*. To study histopathology of the thyroid and parathyroid glands in HIV-infected African Americans in the United States. *Methods*. A retrospective review of 102 autopsy cases done by the Department of Pathology at Howard University Hospital from 1980 through 2007 was conducted. The histopathological findings of the thyroid and parathyroid glands were reviewed, both macroscopically and microscopically. A control group of autopsy patients with chronic non-HIV diseases was examined. *Results*. There were 71 males (70%) and 31 females (30%) with an average age of 38 years (range: 20–71 y). Thirteen patients with abnormal thyroid findings were identified. Interstitial fibrosis was the most common histological finding (4.9%), followed by thyroid hyperplasia (1.9%). Infectious disease affecting the thyroid gland was limited to 2.9% and consisted of mycobacterium tuberculosis, *Cryptococcus* neoformans, and cytomegalovirus. Kaposi sarcoma of the thyroid gland was present in only one case (0.9%). Parathyroid hyperplasia was the most common histological change noted in the parathyroid glands. Comparing the histological findings of cases and controls, we found a similar involvement of the thyroid, with a greater prevalence of parathyroid hyperplasia in HIV patients. *Conclusion*. Thyroid and parathyroid abnormalities are uncommon findings in the HIV-infected African American population.

## 1. Introduction

Human immunodeficiency virus (HIV) and Acquired Immune Deficiency Syndrome (AIDS) are associated with multiple endocrine abnormalities [1]. Several investigators have studied the metabolic derangements and reported the functional abnormalities with specific endocrine glands [2]. There is however scant literature regarding histopathology of the thyroid and parathyroid glands in patients with HIV and AIDS, particularly in African Americans (AA). Nevertheless, extensive literature is available on serum biochemical thyroid functions and calcium fluctuations in these patients. Euthyroid sick syndrome, hypothyroidism, hypocalcemia, impaired parathyroid hormone secretion, and vitamin D deficiency appear to be common among the adult HIV-infected patients [3–5].

We postulated that the thyroid and parathyroid glands would be involved in AIDS patients as evidenced by functional abnormalities and derangements seen in these patients. Our study is the first retrospective report to describe the histopathology of the thyroid and parathyroid glands in HIV-infected AA patients in an inner city hospital in the United States.

## 2. Materials and Methods

A retrospective review of histopathology findings of the thyroid and parathyroid glands at autopsy was conducted at a tertiary care teaching hospital during the year 1980 through 2007. One hundred and two HIV-infected patients who died after admission to Howard University Hospital were identified. The study was reviewed and approved by the Institutional Review Board.

At the time of autopsy, thyroid and parathyroid glands were examined macroscopically and microscopically. Two transverse sections of the thyroid gland were routinely performed. If pathological lesions were noted, multiple sections were performed. Histopathological diagnosis was confirmed by two certified pathologists. Sections were stained with hematoxylin and eosin. Other stains included periodic acid Schiff, Grocott's methenamine silver and Ziehl-Neelsen stain as necessary.

The starting time of the study coincided with the introduction of Highly Active Antiretroviral Treatment (HAART); hence, no many patients were on HAART. Additionally, one third of patients reviewed died before initiation of HAART. We could retrieve a CD4 count only in 20.5% of patients. Incomplete adherence to the prescribed regimen was common, as self-reported by patients as well as evidenced by the number of HIV associated opportunistic infections these patients harbored. We compared the histopathological findings to a control-group which included patients without HIV infection (31 females and 44 males) who died at the hospital from other causes. Chi-square test was used to calculate the significance of these observations.

## 3. Results

### 3.1. Patient Characteristics

One hundred and two autopsy cases were reviewed in this study. There were 71 males (70%) and 31 females (30%) with an average age of 38 (range: 20–71 years). The mean time from HIV diagnosis to death was 53 months (range: 1–144 months). Most of the patients had AIDS, as suggested by the number of opportunistic infections they had (% patients had AIDS defining illnesses). CD4 lymphocyte count was documented in 20.5% of patients with a median CD4 count of 50 cells/*μ*L. Eighty patients (78.4%) did not receive Pneumocystis jiroveci prophylaxis. Thirty-four (33%) of patients were from the pre-HAART period (1980–1990). Fourteen patients were on HAART. Our series included 40 patients with intravenous drug abuse (39.2%), 23 (22.5%) with heterosexual risk, 23 (22.5%) with homosexual or bisexual risk, and 56 (55%) with undocumented sexual preference. It is to be noted that these high risk behavior patterns were not mutually exclusive.

A control group for thyroid histopathologic examination was obtained in 75 non-HIV-infected patients. Comparing the histological findings of cases and controls, we found similar involvement of the thyroid, with greater prevalence of parathyroid hyperplasia in HIV patients.

### 3.2. Pathologic Findings

The thyroid gland weighed between 3 to 40 g. Weights of parathyroid glands were not available. Thyroid gland findings are shown in Table 1. Thirteen patients (7 males and 6 females) had abnormal histopathology of the thyroid gland. The mean age was 41 years, with a range of 30–62 years. Seven of these patients had a history of intravenous drug abuse. The mean time from HIV diagnosis to death was 65.8 months ranging from 6 to 132 months. Only 4 of 13 patients had a CD4 count available with a mean 64, range of 0–200. One patient in this group was receiving Pneumocystis jiroveci prophylaxis and 4 patients were on HAART. The mean body mass index (BMI) was 25 kg/m^2^ with a range of 15–40 kg/m^2^. The mean weight of the thyroid gland was 21.4 grams with a range of 3 to 32 grams.

The gross examination of the thyroid gland was unremarkable in 100 patients (98%). One patient had a macroscopically nodular thyroid gland while another patient had an atrophic thyroid gland. The latter patient had both macroscopic (atrophy) and microscopic abnormalities (fibrosis) (Table 1). In the control group, the macroscopic histopathologic analysis of the thyroid revealed 68 patients (90.6%) having a normal thyroid macroscopic examination (Table 1).

Histological diagnoses are summarized in Tables 3 and 4.

**Figure 1:**
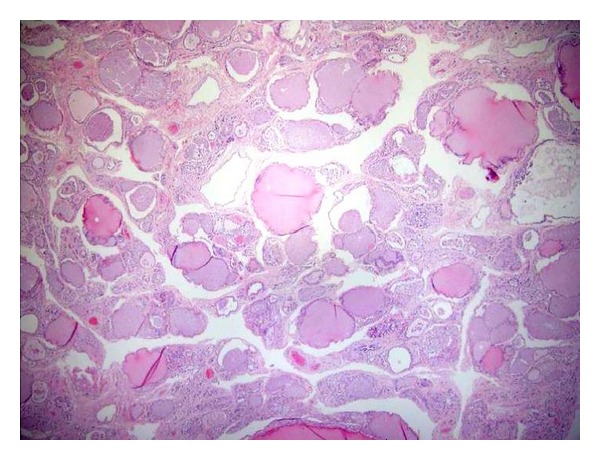
Microscopic section of the thyroid showing fibrosis of the interstitium (H&E, 400x).

Interstitial fibrosis (Figure 1) was the most common histological finding identified in thyroid gland sections (4.9%), followed by thyroid hyperplasia (1.9%). Isolated mild interstitial fibrosis was found in 2 patients. Two patients had interstitial fibrosis associated with hyperplasia of the thyroid gland. One patient showed a moderate degree of fibrosis and atrophy of the thyroid gland. One case of colloid goiter and one case of thyroid adenoma were also identified at autopsy (Table 3).

**Figure 2:**
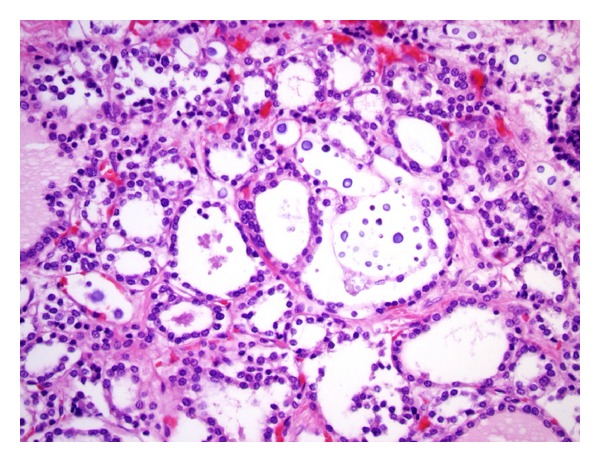
Microscopic section of the thyroid gland, (H&E) showing *Cryptococcus* with variation in size (400x).

**Figure 3:**
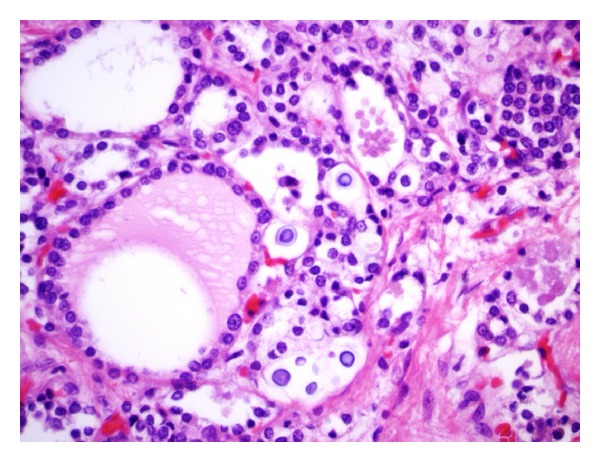
Microscopic section of the thyroid gland, (H&E) showing *Cryptococcus* (600x).

Infections of the thyroid gland included cytomegalovirus (1 case), Mycobacterium tuberculosis (1 case), and *Cryptococcus* (1 case), (Figures 2 and 3).

**Figure 4:**
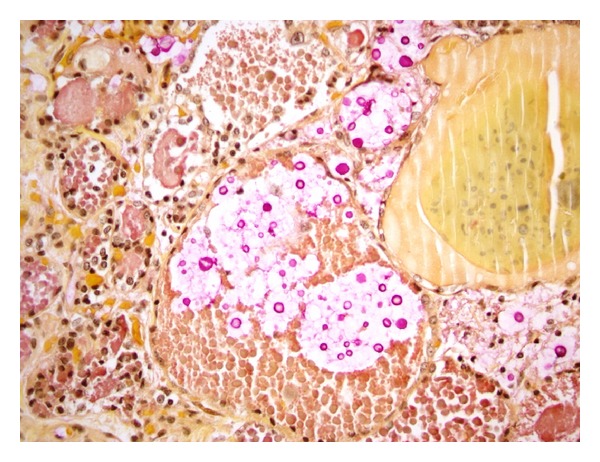
Microscopic section of the thyroid gland, mucin stain highlights yeast with thick capsule (400x).

**Figure 5:**
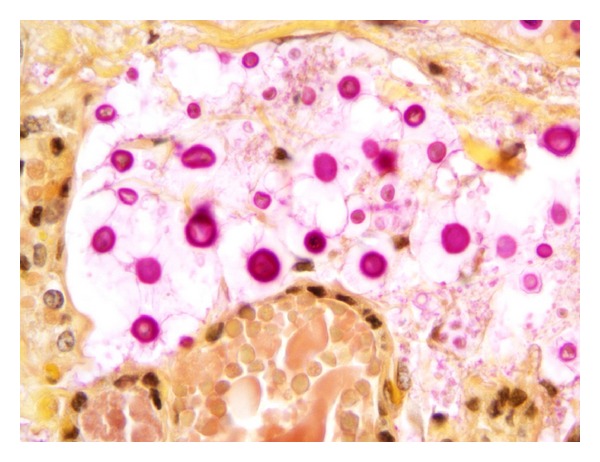
Microscopic section of the thyroid gland, mucin stain demonstrating thick capsule, and pleomorphic yeasts (1000x).

Furthermore, the most common systemic opportunistic infection in 13 of our patients with thyroid abnormalities was Mycobacterium avium complex infection (MAC) (38.4%) followed by Candida albicans (Figures 4 and 5). In the remaining HIV-infected patients without thyroid abnormalities, the most frequent opportunistic infection was Pneumocystis jiroveci (32.5%), followed equally by Candida and cytomegalovirus (19.1%).

**Figure 6:**
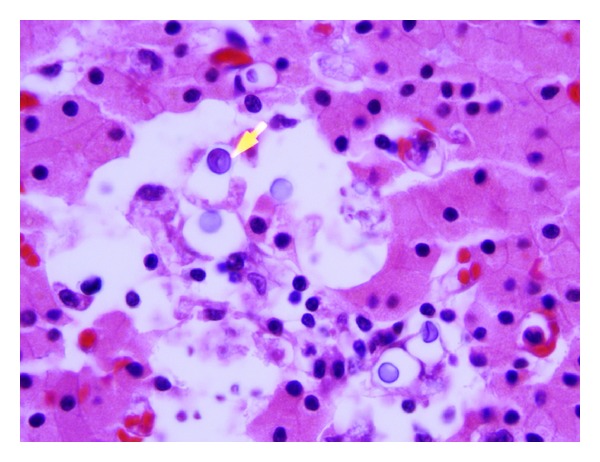
Microscopic section of the parathyroid gland showing *Cryptococcus* (yellow arrow). H&E (1000x).

**Figure 7:**
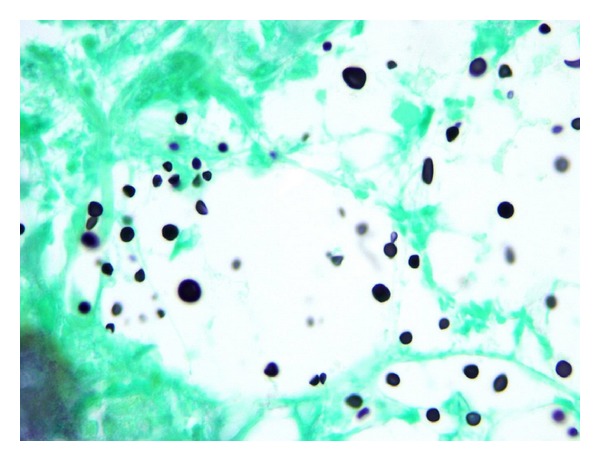
Microscopic section of the parathyroid gland, silver stain highlighting *Cryptococcus* with occasional narrow based budding (1000x).

Parathyroid glands involvement was noted in 32.1% of patients. Parathyroid hyperplasia was by far the most common histological finding accounting for 22.5% of cases followed by cytomegalovirus (CMV) infection of the parathyroid (2.9%) and nodular oncocytic hyperplasia (2.9%). Parathyroid hyperplasia was diagnosed if at least two of all four parathyroid glands were hyperplastic. Fatty infiltration (1.9%) and serous atrophy (1.9%) were also identified in the parathyroid glands (Table 4) (See Figures 6 and 7).

Most of these patients studied died of septic shock or respiratory failure (data not shown).

Review of patient's data showed that abnormal pathological findings were found entirely in patients with ongoing illicit drug use.

The histological findings in control patients revealed cytological appearances consistent with benign thyroid nodular disease in 8% of control patients, interstitial fibrosis in 2.6%, lymphocytic thyroiditis in 2.6%, cryptococcal infection in 1.3%, papillary carcinoma in 1.3%, and mycobacterial tuberculosis in 1.3% of the control specimens (Table 3). The histological appearance of parathyroid glands from the control group did differ from the HIV group with 72 control patients (96%) showing normal histological appearance of the parathyroids as opposed 69 HIV-infected patients (67.6%) (Table 2).

## 4. Discussion

HIV infection and antiretroviral therapy can induce endocrine dysfunction. Patients with AIDS have increased prevalence of nonthyroidal illness, hypothyroidism, and abnormal serum parathyroid hormone (PTH) and serum calcium levels [4, 5]. These alterations in thyroid hormones and calcium homeostasis are rarely the result of a direct infection or infiltration of the thyroid and parathyroid glands. Although subclinical hypothyroidism has been recognized as more prevalent among HIV-infected individuals, it does not appear to have an autoimmune basis [6]. Graves's disease subsequent to immune restoration due to HAART has been described and unlike the common infection-related immune reconstitution syndromes, it is usually diagnosed 12–36 months after HAART initiation [7]. Two studies from South America have described pathological changes in the thyroid gland in AIDS patients [8, 9]. However, no investigator has reported the histopathology of parathyroid glands in Human Immunodeficiency Virus (HIV) patients. This study represents the first detailed report of thyroid and parathyroid gland abnormalities in a HIV-infected African-American population in the United States.

Ethnicity-related differences in organ systems involvement in HIV patients have been described previously by Morgello et al. with cachexia, renal, cardiac and splenic involvement more frequent in blacks than in whites and/or Hispanic individuals [10]. Additionally, *Mycobacterium avium-intracellulare* (MAI) infection is also more commonly seen in blacks than in whites and/or Hispanic individuals [11]. However the exact explanation for these discrepancies is not clear.

Our findings are strikingly different from what have been published so far in terms of the frequency of thyroid involvement in HIV.

Basílio-De-Oliveira from Brazil reviewed autopsy cases of 100 AIDS patients [8]. The study included 72 white patients. Compared to our findings, thyroid involvement by infectious processes was significant. Mycobacterium tuberculosis infection of the thyroid gland was found in 23% of patients and cytomegalovirus (CMV) in 17%. Neoplastic involvement of the thyroid was also higher in frequency with Kaposi sarcoma (2%) and occult papillary carcinoma (4%) seen in patients. Histopathological lesions consisted mainly of interstitial fibrosis with follicular atrophy. Lima et al. studied forty-seven thyroids obtained at autopsy from 38 men and 9 women with AIDS in Brazil [9]. However, the ethnicity of the sample population was not documented. In contrast to our results, they identified greater frequency of infectious pathogens (14 cases, 29.7%) with five cases of mycobacterial infection (10.6%), four cases of histoplasmosis and cryptococcosis, and finally one case of paracoccidioidomycosis [11]. Their results were concordant with Basílio-De-Oliveira in regard to Mycobacterium infection being the most frequently detected agent. This may be due to the higher prevalence of mycobacteria in AIDS patients in Brazil [11, 12].

In postmortem examinations of these patients, thyroid pathology was common affecting 29 patients (61.3%), with nonspecific focal chronic inflammation affecting 14 cases (48.2%), colloid goiter in 5 cases (17.2%), and lipomatosis in 4 cases (13.7%). Lipomatosis was associated with atrophy (1 case), hyperplastic nodule (1 case), and histoplasmosis (1 case) [9].

In our study, the frequency of infectious etiology affecting the thyroid gland was limited to 2.9% (3 cases).There was only one case of cytomegalovirus (CMV), *Cryptococcus*, and tuberculosis. All our cases inclined to have occurred in the context of a widely 5 disseminated disease.

Several cases have been previously reported also as part of multiple organ involvement either antemortem or at autopsy [13–17]. In our series, some patients presented with multiple coexisting opportunistic infections. About 45% of patients in the HIV group with thyroid pathology had more than 2 opportunistic infections as opposed to 35% in the subgroup of HIV patients with normal thyroid histopathology (Table 5). Based on the limited number of patients, we cannot postulate a possible association between opportunistic infections and the occurrence of thyroid abnormalities.

While in previous case reports, Pneumocystis jiroveci has been a prominent cause of thyroiditis in HIV patients [18–21], particularly in patients on aerosolized pentamidine [22, 23], we did not identify this microorganism in any of our patients. This may be due to the fact that many patients presented to our hospital with symptoms suggestive of Pneumocystis jiroveci pneumonia and were treated promptly with trimethoprim-sulfamethoxazole leading to absence of histological evidence of this microorganism at autopsy.

This observation is consistent with other studies showing that after “curing” patients of their pneumocystis infections, biopsy specimens usually lack evidence of residual disease [24, 25].

Our most common finding was interstitial fibrosis seen in 5 of the 12 microscopic cases. Contempre et al. have linked thyroid fibrosis to transforming growth factor beta (TGF-beta) in which follicular cell necrosis occurs first followed by thyroid fibrosis in the setting of selenium deficiency [26]. Similarly, interstitial fibrosis of the thyroid gland in our series could represent the histologic sequelae of previous inflammatory or infectious assaults coupled with an impaired tissue repair due to the underlying immunosuppression. In addition, Human Immunodeficiency Virus infection itself is associated with increased levels of transforming growth factor beta (TGF-beta) [27].

Of note, we identified two cases of thyroid hyperplasia which has been described as a normal response to alterations in the feedback mechanism of thyrotropin-releasing hormone and thyroid-stimulating hormone [28]. Additionally, it has been shown that HIV-1-infected inflammatory cells may release a mitogen protein (TAT) which enhances the production of growth factors including fibroblast growth factor (FGF-1 and FGF-2) and transforming growth factor-beta [29]. These fibroblast growth factors appear to be involved in the pathogenesis of thyroid hyperplasia [30].

Neoplastic involvement of the thyroid gland was present in one case. Kaposi sarcoma is the most common malignancy associated with HIV infection [31]. Kaposi sarcoma of the thyroid is uncommon, described only in the context of a widespread metastasis [32]. Its pathogenesis involves immunodeficiency, oncogenic DNA viruses, and the HIV-1 protein Tat [33].

Little is known about the relative contribution of illicit drugs use to the thyroid histopathology in HIV-infected populations. Our study suggests that ongoing drug use may impact thyroid tissue in HIV patients.

There is scant literature about the histopathology of the parathyroid glands in HIV patients. We identified parathyroid hyperplasia as the most common histological process (Table 4). The histologic appearance of parathyroid hyperplasia was hypercellularity with heterogenous cell proliferation but predominantly chief cells associated with reduced stromal fat and involving more than one gland.

This finding could be reflective of the secondary hyperparathyroidism resulting from the high prevalence of vitamin D deficiency in African American [34] in general and also specifically HIV-infected individuals [35, 36], although we do not have vitamin D levels in any of these patients. Both parathyroid and nodular oncocytic hyperplasia have been described as a feature of secondary hyperparathyroidism [37]. In the HIV population in particular, this process can also result from decreased serum calcium secondary to impairment in renal function, nutritional status, and chronic malabsorption.

Of note, a decrease of parathyroid hormone (PTH) level has also been previously reported in human immunodeficiency virus (HIV)-infected patients [38]. The mechanism might be related to antibodies against parathyroid cells. Using anti-Leu3a, a monoclonal antibody recognizing CD4, HIV-positive patients have been found to express CD4 molecule at the surface of parathyroid gland cells, indicating the possibility of either functional inhibition by anti-CD4 antibodies or direct infection by HIV [39].

In addition, current evidence indicates that HIV-infected persons have micronutrient deficiencies [40]. Therefore, the question of whether parathyroid hyperplasia could be related to possible micronutrient deficiency such as iodine deficiency remains to be established in HIV-infected subjects. Further research is needed to elucidate the role of micronutrient deficiencies on parathyroid pathology in HIV-infected subjects.

Our study findings must be interpreted in light of several limitations. First, our findings stem from a retrospective review of general autopsies in HIV-infected African American patients; hence, the small number of histologic sections might have influenced the microscopic findings recorded.

Larger studies focusing on thyroid and parathyroid are required to establish the prevalence of our findings.

Second, we were unable to analyze the thyroid function tests and the autoimmune status of our sample. One could argue that interstitial fibrosis is fairly non-specific and further prospective studies correlating histopathological findings with thyroid serologies for a better assessment of thyroid pathology in HIV African American population are needed.

## 5. Conclusion

We conclude that thyroid and parathyroid abnormalities are uncommon findings in the HIV-infected African American population. The most common characteristics of histopathology seen in the thyroid and parathyroid glands in these patients include interstitial fibrosis and parathyroid hyperplasia, respectively.

## Figures and Tables

**Table 1:** Summary of thyroid gland findings in HIV-infected patients and controls.

Thyroid*	HIV-infected patients *N* (%)	Non-HIV-infected patients *N* (%)	*P* value
Normal	89 (87.3)	65 (86.6)	ns
Abnormal (microscopic)	12 (11.8)	10 (13.3)	0.11
Abnormal (macroscopic)	2 (1.96)	7 (9.3)	0.05

*The numbers will not sum to 100% since the categories are not mutually exclusive.

**Table 2:** Summary of parathyroid glands findings in HIV-infected patients and controls.

Parathyroids**	HIV-infected patients *N* (%)	Non-HIV-infected patients *N* (%)	*P* value
Normal	69 (67.6)	72 (96)	ns
Abnormal (microscopic)	33 (32.3)	3 (4)	0.04
Abnormal (macroscopic)	2 (1.96)	2 (2.6)	0.96

**The numbers will not sum to 100% since the categories are not mutually exclusive.

**Table 3:** Histopathological findings in thyroid gland.

Histologic findings	Frequency HIV-infected patients (%)	Frequency Non-HIV-infected patients (%)
Nodular goiter	3 (2.7%)	6 (8%)
Cryptococcal infection	1 (0.9%)	1 (1.3%)
Mycobacterium Tuberculosis	1 (0.9%)	1 (1.3%)
Kaposi sarcoma	1 (0.9%)	0
CMV infection	1 (0.9%)	0
Interstitial fibrosis	5 (4.9%)	2 (2.6%)
Lymphocytic thyroiditis	0	2 (2.6%)
Papillary carcinoma	0	1 (1.3%)

**Table 4:** Histopathological findings in parathyroid glands.

Histologic findings	Frequency HIV-infected patients (%)	Frequency Non-HIV-infected patients (%)
Parathyroid hyperplasia	23 (22.5%)	2 (2.6%)
Nodular oncocytic hyperplasia	3 (2.9%)	0
CMV infection	3 (2.9%)	0
Fatty infiltration	2 (1.9%)	1 (1.3% )
Serous atrophy	2 (1.9%)	0

CMV: cytomegalovirus.

**Table 5:** Frequency of opportunistic infections in HIV infected individuals.

Number of opportunistic infections	Patients with normal thyroid glands *n* = 89 (%)	Patients with abnormal thyroid glands *n* = 13 (%)
0	30 (33.7%)	1 (7.7%)
1	27 (30.3%)	6 (46.1%)
2	21 (23.5%)	4 (30.7%)
3	7 (7.8%)	2 (15.3%)
4	2 (2.2%)	0
5	1 (1.1%)	0
6*	1 (1.1%)	0

*MAC, cytomegalovirus, *Cryptococcus*, Candida, Pneumocystis jiroveci, and Herpes.

## Abbreviations

HIV: Human Immunodeficiency Virus
AIDS: Acquired Immune Deficiency Syndrome
HAART: Highly Active Antiretroviral Treatment
BMI: Body mass index
CMV: Cytomegalovirus
MAI: *Mycobacterium avium-intracellulare*
PTH: Parathyroid hormone
MAC: Mycobacterium avium complex
TGF-beta: Transforming growth factor beta
FGF: Fibroblast growth factor
H&E: Hematoxylin and eosin stain.
